# Allosteric Modulation of GSK-3β as a New Therapeutic Approach in Limb Girdle Muscular Dystrophy R1 Calpain 3-Related

**DOI:** 10.3390/ijms22147367

**Published:** 2021-07-08

**Authors:** Anabel Rico, Garazi Guembelzu, Valle Palomo, Ana Martínez, Ana Aiastui, Leire Casas-Fraile, Andrea Valls, Adolfo López de Munain, Amets Sáenz

**Affiliations:** 1 Biodonostia Health Research Institute, Neurosciences Area, 20014 San Sebastian, Spain; anabelr6@gmail.com (A.R.); garazi13@gmail.com (G.G.); leire.casas@kuleuven.be (L.C.-F.); andrea.valls@biodonostia.org (A.V.); adolfojose.lopezdemunainarregui@osakidetza.eus (A.L.d.M.); 2 Spanish Ministry of Economy & Competitiveness, Carlos III Health Institute, CIBERNED, 28031 Madrid, Spain; vpalomo@cib.csic.es (V.P.); ana.martinez@csic.es (A.M.); 3 Centro de Investigaciones Biológicas Margarita Salas CSIC, 28040 Madrid, Spain; 4 Biodonostia Health Research Institute, Cell Culture and Histology Platform, 20014 San Sebastian, Spain; ana.aiastui@biodonostia.org; 5 Osakidetza Basque Health Service, Donostialdea Integrated Health Organisation, Department of Neurology, 20014 San Sebastian, Spain; 6 Department of Neurosciences, University of the Basque Country UPV-EHU, 20014 San Sebastian, Spain

**Keywords:** LGMDR1, limb-girdle muscular dystrophy, *CAPN3*, tideglusib, VP0.7, GSK-3β, Wnt, mTOR

## Abstract

Limb-girdle muscular dystrophy R1 calpain 3-related (LGMDR1) is an autosomal recessive muscular dystrophy produced by mutations in the *CAPN3* gene. It is a rare disease and there is no cure or treatment for the disease while the pathophysiological mechanism by which the absence of calpain 3 provokes the dystrophy in muscles is not clear. However, key proteins implicated in Wnt and mTOR signaling pathways, which regulate muscle homeostasis, showed a considerable reduction in their expression and in their phosphorylation in LGMDR1 patients’ muscles. Finally, the administration of tideglusib and VP0.7, ATP non-competitive inhibitors of glycogen synthase kinase 3β (GSK-3β), restore the expression and phosphorylation of these proteins in LGMDR1 cells, opening the possibility of their use as therapeutic options.

## 1. Introduction

Limb-girdle muscular dystrophy R1 calpain 3-related (LGMDR1) is an autosomal recessive muscular dystrophy produced by mutations in the *CAPN3* gene that causes progressive degeneration of the proximal muscles of the pelvic and shoulder girdle. The disease begins in the second decade of life and muscle degeneration leads to muscle weakness and atrophy that confines patients to a wheelchair in around 20 years of disease progression [1,2]. Subsequently, as muscle degeneration progresses, it becomes a highly disabling disease that prevents patients from performing simple daily tasks. Unfortunately, to date, there is no therapy that cures or even slows down the progression of muscle fiber degeneration.

Calpain 3 is a muscle-specific protease that may participate in several functions, such as muscle contraction due to its link to titin [3,4,5,6,7], cell membrane homeostasis [8,9] and the regulation of Ca^2+^ flow between the sarcoplasmic reticulum/cytoplasm [10].

Balanced homeostasis between the synthesis and degradation of proteins in the muscle fiber is key to maintain the muscle and thus to avoid muscle atrophy and weakness [11]. For that purpose, there are certain signaling pathways, such as the Akt/mTOR or the Wnt signaling pathways, which stimulate protein synthesis, myofiber growth and inhibit protein degradation [12]. They also participate in differentiation during muscle development and in the regeneration of muscle fiber in adults [13].

When the Wnt signaling pathway is active, Wnt ligands induce the inactivation of GSK-3β preventing β-catenin phosphorylation, allowing its accumulation in the cytoplasm and translocating it to the nucleus. Then, β-catenin binds to T-Cell Factor/Lymphoid Enhancer Factor (TCF/LEF) and activates downstream target genes [14,15]. On the contrary, when the Wnt signaling pathway is inactive, GSK-3β is activated. It phosphorylates β-catenin so that it is subsequently degraded [16]. GSK-3β is a constitutively active kinase that controls numerous aspects of cell physiology, such as proliferation, metabolism and apoptosis [17,18,19,20].

Among the drugs that inhibit GSK-3β, lithium is a widely used drug. Due to its activator role in the Wnt signaling pathway, certain studies showed beneficial results in vitro [21,22,23,24]. Additionally, in vivo studies have shown protective effects in a slowly progressive spinal muscle atrophy mouse model [25] and improvement in muscle size and strength in an LGMD1D preclinical mouse model [26].

Among the molecules that are able to inhibit GSK-3, the ATP-competitive ones have often presented important adverse side effects in long-term treatments. On the other hand, those that inhibit GSK-3β in a non-competitive or allosteric way are more selective [27,28,29], with the thiadiazolidinone (TDZD) family being the first ATP non-competitive inhibitor of GSK-3β reported. Since then, various highly selective and allosteric analog drugs were synthesized, including tideglusib and VP0.7 [27,30].

Tideglusib is an irreversible drug designed for the treatment of Alzheimer’s disease and whose safety for human treatment has been demonstrated [31]. VP0.7, on the other hand, is a drug that modulates the kinase allosterically [30]. Furthermore, it has been reported that a VP0.7 and a second structural related derivative correct delayed myogenesis in myoblasts from patients with type 1 congenital myotonic dystrophy (CDM1) [27].

The pathophysiological mechanism by which the absence of calpain 3 provokes the dystrophy in muscles is not clear. Loss of calpain 3 leads to the deregulation of the expression of several genes/proteins and to abnormal sarcomere formation in the muscles [24,32,33]. Costameres are complexes that may rule the sarcomere assembly and stabilization [34,35,36]. They enable the adhesion between the sarcomere in the muscle and the extracellular matrix and this linkage is partially mediated by integrins [37,38]. In LGMDR1 myotubes, the physiologically required replacement of the integrin β1D isoform is disturbed and may be the cause of incorrect costamere assembly. Moreover, a crosstalk was identified between integrin and Wnt signaling pathways [24]. Currently, there is no cure or treatment for limb girdle muscular dystrophy R1 calpain 3-related.

In this work, we report expression alterations in proteins implicated in signaling pathways that regulate muscle homeostasis, such as Wnt and mTOR pathways. LGMDR1 patients’ muscles showed a severe reduction in the expression of the proteins involved in these pathways. Finally, our study showed that tideglusib and VP0.7, ATP non-competitive GSK-3β inhibitors, restore the expression and phosphorylation of key proteins in Wnt and mTOR pathways, opening up the possibility of their use as therapeutic options in LGMDR1.

## 2. Results

### 2.1. The Wnt/β-Catenin Pathway Is Altered in the Muscle of LGMDR1 Patients

Previous studies had described the overexpression of FRZB, a Wnt1, 5a, 8 and 9a antagonist, in the muscle of LGMDR1 patients [24]. Due to the excessive inhibition of this pathway, the expression of some proteins participating in the Wnt pathway has been analyzed.

**Figure 1:**
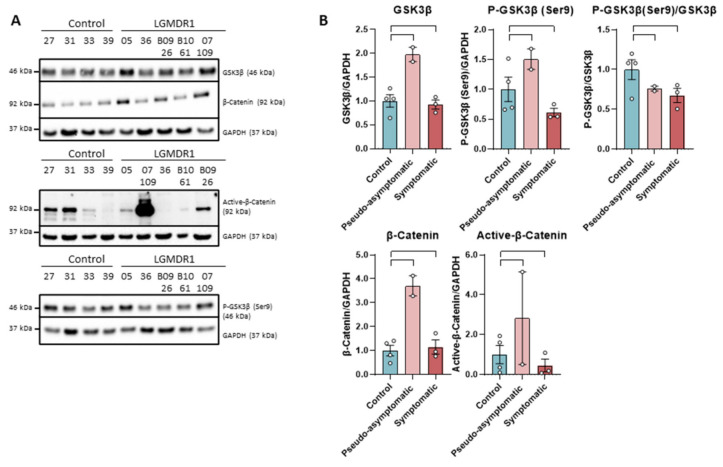
Western blot (**A**) and densitometry analysis (**B**) of Wnt signaling pathway proteins in samples from muscles of LGMDR1 and controls: total GSK-3β, P-GSK-3β (Ser9), P-GSK-3β/GSK-3β, β-catenin and Active β-catenin. Control samples (*n* = 4), symptomatic patients (*n* = 3) and pseudo-asymptomatic patients (*n* = 2) (05 and 07-109). All error bars represent standard error of the mean (SEM). Each circle represents a sample. Loading control: GAPDH.

The levels of GSK-3β and total β-catenin do not show differences between patients and controls, as well as p-GSK-3β (Ser9). On the contrary, the active form of β-catenin showed a drastic decrease of the protein in patients, except in a benign patient (B09-26). This reduction was not observed in the pseudo-asymptomatic cases (05 and 07-109). These pseudo-asymptomatic cases were 13- and 10-year-old patients who only presented hyperCKemia and eosinophilia. In most of the analyzed proteins, an increase in expression (not statistically significant, *n* = 2) has been observed in pseudo-asymptomatic cases with respect to the controls (Figure 1).

### 2.2. Reduction in the Activation of the mTOR Pathway in the Muscle of LGMDR1 Patients

**Figure 2:**
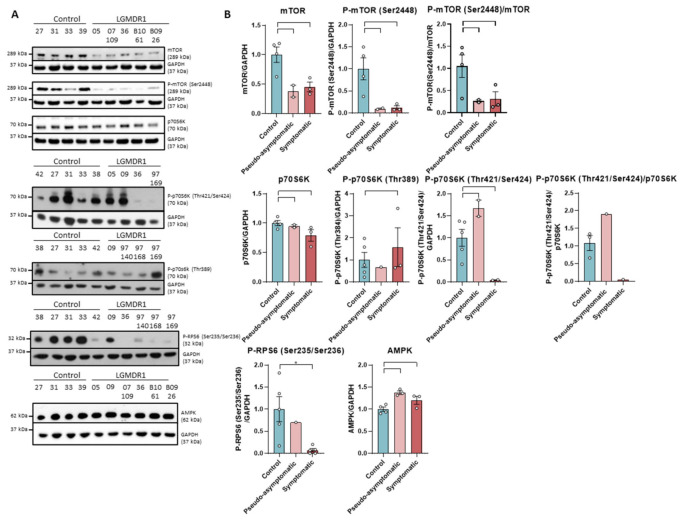
Western blot (**A**) and densitometry analysis (**B**) mTOR signaling pathway proteins in samples from muscles of LGMR1 and controls: mTOR, P-mTOR (Ser2448), mTOR/P-mTOR (Ser2448), p70S6K, P-p70S6K (Thr389), P-p70S6K (Thr421/Ser424), p70S6K/P-p70S6K (Thr421/Ser424), P-RPS6 (Ser235/Ser236) and AMPK. P-RPS6 is statistically significant in symptomatic patients (*p* = 0.0159). 05, 09 and 07-109: pseudo-asymptomatic patients. *Control samples* (*n* = 4) for mTOR, P-mTOR, p70S6K and AMPK and (*n* = 5) for P-p70S6K -Thr389 and Thr421/Ser424- and P-RPS6. *Symptomatic patients* (*n* = 3) for mTOR, P-mTOR, p70S6K, P-p70S6K (Thr389), P-RPS6 and AMPK, (*n* = 2) for P-p70S6K (Thr421/Ser424) and (*n* = 1) for p70S6K/P-p70S6K (Thr421/Ser424). *Pseudo-asymptomatic patients* (*n* = 3) for AMPK, (*n* = 2) for mTOR, P-mTOR, p70S6K and P-p70S6K (Thr421/Ser424) and (*n* = 1) for P-p70S6K (Thr389), P-RPS6. All error bars represent standard error of the mean (SEM). *: Statistically significant. Each circle represents a sample. Loading control: GAPDH.

The expression of mTOR as well as its phosphorylated form in Ser2448 are severely reduced in the muscle of LGMDR1 patients. The decrease of both forms can be observed in pseudo-asymptomatic and symptomatic patients (Figure 2).

Subsequently, the expression of kinases downstream of this pathway was analyzed. p70S6K is a kinase participating in the mTOR pathway that is activated by sequential phosphorylations [39,40,41]. Our results show that the levels of total protein p70S6K and its phosphorylated form at residue Thr389 in controls and patients are similar. In contrast, symptomatic patients showed a greatly decreased level of phosphorylation at the Thr421/Thr424 residues (Figure 2). Downstream, the kinase p70S6K catalyzes the phosphorylation of ribosomal protein S6 (RPS6), whose phosphorylation correlates with high levels of protein translation and synthesis [40,42]. When analyzing mTOR, P-mTOR (Ser2448) and PRPS6 (Ser235/Ser236), a statistically significant decrease was found in the muscle of LGMDR1 patients when both subgroups were analyzed together (*p* = 0.00159; *p* = 0.00159 and *p* = 0.0303, respectively, data not shown).

On the other hand, as it is known that AMPK kinase is an inhibitor of the mTOR pathway [43,44,45], it was analyzed in the muscle of the patients, and an increase in the amount of this kinase was observed in the group of patients (*p* = 0.0381) (Figure 2).

**Figure 3:**
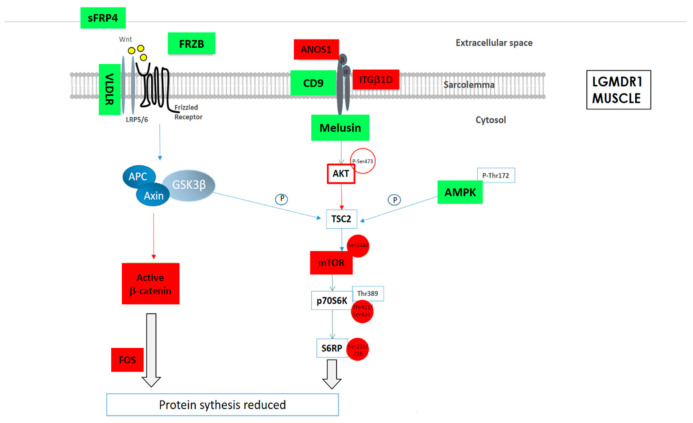
Schematic representation of the altered protein expression or phosphorylations in Wnt and mTOR signaling pathways in LGMDR1 muscle. Green: Upregulated expression or phosphorylation in LGMDR1. Red: Downregulated expression in LGMDR1.

As a summary, a schematic representation of the altered proteins in Wnt and mTOR pathways in LGMDR1 muscle are shown (Figure 3).

### 2.3. In Vitro Treatment with GSK-3β Inhibitors: Tidelgusib, VP0.7 and Li

Lithium and the small molecule drugs tideglusib and VP0.7 were used in this study. While lithium is a known and widely used GSK-3 inhibitor, its unwanted adverse effects entailed in clinical trials led us to the search of selective GSK-3 inhibitors with an optimal safety profile, including tideglusib and VP0.7. These drugs were administered in different cell types: primary myotubes, CD56- cells and skin fibroblasts. The effect on the response for the administered drugs was different depending on the cell type.

#### 2.3.1. Activation of the Wnt Pathway in Human Myotubes and Recovery of Deregulated Gene Expression in LGMDR1 Patients

**Figure 4:**
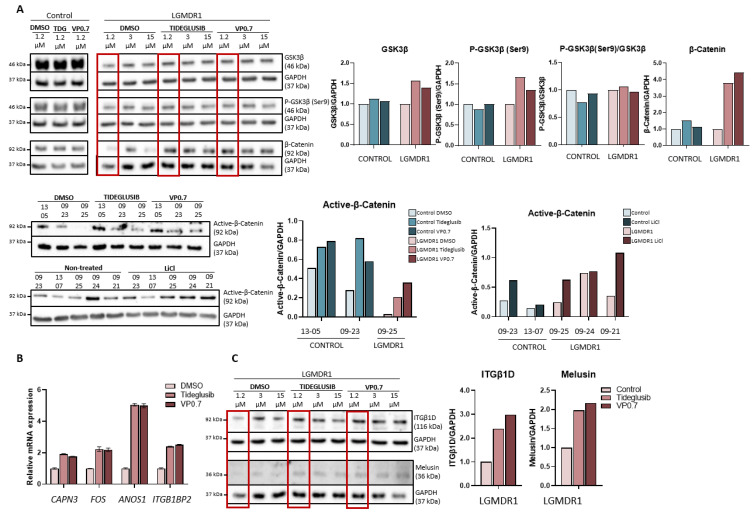
Activation of the Wnt pathway in human myotubes on day 8 of differentiation and after 48 h of treatment with Tideglusib and VP0.7 at three concentrations (1.2, 3.0 and 15.0 µM) and LiCl in control (*n* = 1) and patient LGMDR1 (*n* = 1). (**A**) Wnt signaling pathway activation: western blot of total GSK-3β, P-GSK-3β (Ser9), P-GSK-3β/GSK-3β, total β-catenin and active β-catenin. (**B**) Expression of *CAPN3*, *FOS*, *ANOS1* and *ITGB1BP2* genes in LGMDR1 myotubes (*n* = 1 with technical triplicates). (**C**) Western blot of structural proteins: ITGβ1D and melusin. In red, lowest concentration used in the LGMDR1 myotubes (1.2 µM) for densitometry analysis.

We proceeded to analyze the expression of GSK-3β, as well as its downstream effect on the Wnt pathway in myotubes from controls and patients after drug administration. Inhibition by tideglusib and VP0.7 slightly increases the amount of total GSK-3β and its phosphorylation (Ser9) in patients. The activation of the Wnt pathway was confirmed by effective inhibition of GSK-3β, since an increase in total β-catenin expression as well as active β-catenin was observed with tideglusib and VP0.7. As a control assay, lithium was administered, and an increase in active β-catenin was also verified (Figure 4A).

On the other hand, in previous studies, we had already described that activating the Wnt pathway through the silencing of the *FRZB* gene or with treatment with lithium, the expression of some altered genes in the muscle of patients was recovered [24,46]. Thus, their expression was analyzed after administering tideglusib and VP0.7. The results showed a correlation with the effect generated by the *FRZB* gene silencing, that is, an increase in the expression of the *CAPN3*, *FOS* and *ANOS1*, and an increase in *ITGB1BP2* was also observed (Figure 4B).

The expression of some of the structural proteins that are part of the costamere, whose expression is altered in LGMDR1 patients, was analyzed. After drug administration, an increase in integrin β1D and melusin was observed (Figure 4C).

#### 2.3.2. Activation of the Akt/mTOR Pathway in Myotubes from LGMDR1 Patients

**Figure 5:**
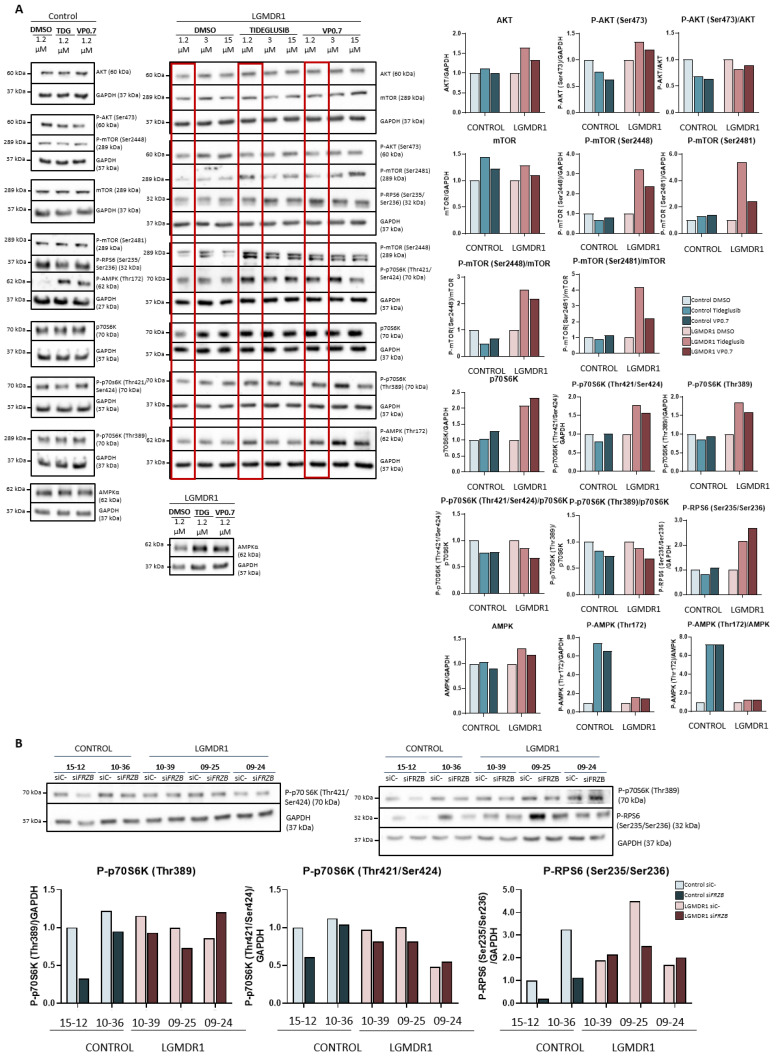
Activation of the Akt/mTOR pathway in human myotubes on day 8 of differentiation and after 48 h of treatment with tideglusib and VP0.7 at three concentrations (1.2, 3.0 and 15.0 µM) in control (*n* = 1) and patient LGMDR1 (*n* = 1): (**A**) western blot of AKT, P-AKT (Ser473), mTOR, P-mTOR (Ser2448 and Ser2481), p70S6K, P-p70S6K (Thr421/Ser424), P-p70S6K (Thr389), P-RPS6 (Ser235/Ser236) and P-AMPK (Thr172). (**B**) Effect of *FRZB* silencing on the mTOR pathway in controls’ (*n* = 2) and LGMDR1 patients’ (*n* = 3) myotubes. The red squares in the WB show the lowest concentration used in patients (1.2 µM) for densitometry analysis. For the quantification of total AMPKα in LGMDR1 patient’s myotubes, only the lowest dose was analyzed.

After administration of the drugs in the patient’s myotubes, the amount of Akt expression and its phosphorylation (Ser473) increased slightly. For mTOR, hardly any changes were observed in total mTOR, but there was a substantial increase in phosphorylation of mTOR in two of its residues (Ser2448 and Ser2481) (Figure 5A).

Downstream of the mTOR pathway, the p70S6K and RPS6 proteins were analyzed. After administration of the drugs, the expression of total p70S6K, its phosphorylations (Thr421/Ser424 and Thr389), as well as the levels of phosphorylation of the RPS6 protein (Ser235/Ser236) increased in the patient’s sample. In the control, the effect observed after the treatments is very mild (Figure 5A).

Finally, AMPK was analyzed after drug administration, and a slight increase in Thr172 residue phosphorylation was observed in the patient and a very marked increase in the control (Figure 5A).

On the other hand, given that in previous studies, the silencing of the *FRZB* gene had shown recovery in the expression of various altered genes in patients, we proceeded to analyze its possible effect beyond the Wnt pathway, specifically in the mTOR pathway. The silencing of the *FRZB* gene carried out in the myotubes did not show any effect on the regulation of the expression or on the phosphorylation of mTOR (data not shown). In addition, the activations of the downstream kinases of this pathway, P-p70S6K (Thr389), P-p70S6K (Thr421/Ser424) and P-RPS6 (Ser235/Ser236) were analyzed, but little effect or a slight decrease was observed (Figure 5B).

#### 2.3.3. Absence of Activation of the Wnt Pathway in Fibroblasts and CD56- after Treatment with GSK-3β Inhibitor Drugs

In order to establish whether the effects previously observed after drug administration in myogenic cells (myoblasts differentiated to myotubes) occurred similarly in other cell types, we proceeded to study skin fibroblasts and CD56- cells obtained from muscle patients. We used two non-myogenic cell lines (one from non-muscular tissue and the other from muscular tissue, but not myogenic) to show that these drugs may not affect other cell lineages.

- Fibroblasts

**Figure 6:**
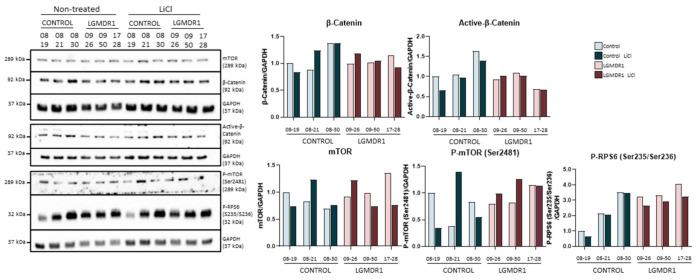
Analysis of fibroblasts of controls (*n* = 3) and LGMDR1 patients (*n* = 3) treated with LiCl.

Treatment with lithium in fibroblasts showed increased phosphorylation of GSK-3β (Ser9), confirming that the treatment produces the desired kinase inhibition (Figure S1). Subsequently, the amount of total β-catenin and its active form were analyzed, as a downstream effect of the activation of the Wnt pathway, but no modification was detected in the expressed amount (Figure 6). Given that GSK-3β can interact with the mTOR pathway, we proceeded to analyze the effect that its inhibition could produce on its regulation. The analysis carried out does not show conclusive results that could suggest any modification in the expression of mTOR or in its phosphorylation (Ser2481). Finally, the phosphorylation of RP6S (Ser235/Ser236) did not show considerable variation after treatment with lithium (Figure 6). Thus, lithium treatment does not seem to activate the Wnt pathway or the mTOR pathway in fibroblasts.

**Figure 7:**
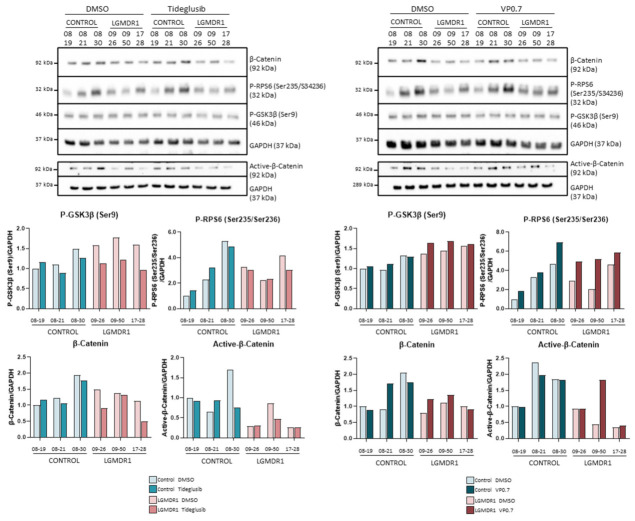
Analysis of fibroblasts of controls (*n* = 3) and LGMDR1 patients (*n* = 3) treated with the GSK-3 inhibitors, tideglusib and VP0.7.

Treatment with tideglusib and VP0.7 did not show an increase in the phosphorylation of GSK-3β (Ser9) (Figure 7). Since they are allosteric inhibitors, their inhibition could not be demonstrated by phosphorylation of Ser9 and it was considered that the inhibition of GSK-3β was taking place. β-catenin and its active form did not show an increase, indicating that the Wnt pathway is not activated in this cell type by means of tideglusib or VP0.7 (Figure 7). Finally, we analyzed the effect that the inhibition of GSK-3β could cause in the mTOR pathway. The phosphorylation of RPS6, only showed a slight increase with the treatment with VP0.7 (Figure 7).

- CD56- cells

**Figure 8:**
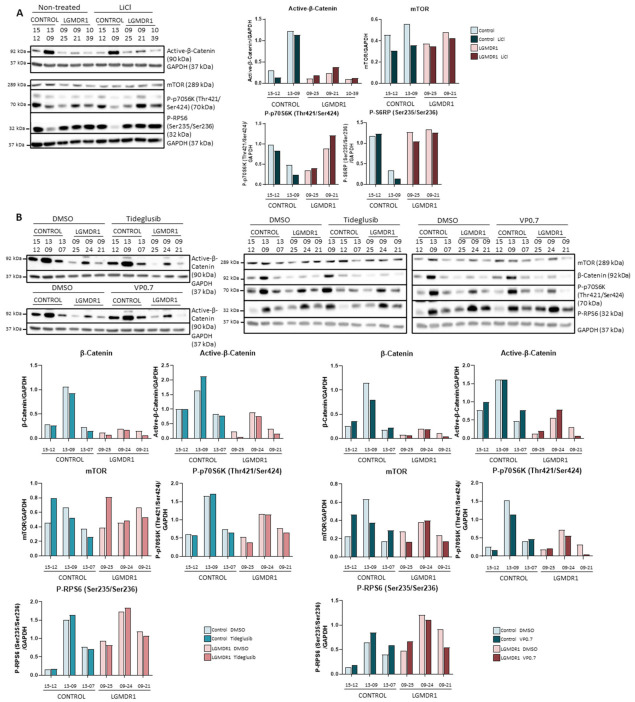
Analysis of CD56- cells treated with (**A**) LiCl (controls, *n* = 2 and LGMDR1 patients, *n* = 3) and (**B**) Tideglusib and VP0.7 (controls, *n* = 3 and LGMDR1 patients, *n* = 3).

As expected, the administration of lithium to CD56- cells showed that inhibition of GSK-3β through phosphorylation of the Ser9 residue occurred, since the amount of phosphorylated GSK-3β was increased (Figure S2). On the contrary, as observed in fibroblasts, no activation of the Wnt pathway was observed, since no difference was detected in the amount of active β-catenin (Figure 8A). The effect that this could have on the regulation of the mTOR pathway was also analyzed. However, no differences were observed downstream of this pathway due to lithium treatment (Figure 8A).

In the same manner, after treatment with tideglusib and VP0.7 in CD56- cells, no substantial alteration was observed in the amount of total β-catenin or in its active form (Figure 8A). Finally, the effect of this inhibition on the regulation of the mTOR pathway was analyzed, but no differences were observed in mTOR or in the phosphorylations of p70S6K and RPS6 (Figure 8A). Thus, the inhibition of GSK-3β by lithium, tideglusib or VP0.7 does not activate the Wnt or mTOR pathways in fibroblasts or CD56- cells (Figure 8B).

## 3. Discussion

It is known that as a consequence of the primary deficiency of a specific protein, there are alterations in different signaling pathways, as occurs in various forms of muscular dystrophy, where the signaling pathways of NF-κB, MAPK, PI3K/Akt and Cn/NFAT are altered [47]. It had previously been described that in the muscle of LGMDR1 patients, the primary deficiency of calpain 3 leads to an increase in NF-κB [48] and dysregulation of gene and protein expression in the Wnt pathway [24,33].

In this work, two signaling pathways that regulate homeostasis in muscle fiber in LGMDR1 patients have been analyzed to establish, first, the pathways with the greatest impact on the pathophysiology of the disease, and second, the effect of the treatment with GSK-3β inhibitor drugs to regulate signaling pathways in patients.

### 3.1. Alteration of the Wnt and Akt/mTOR Pathways

Our study has allowed to establish that in the muscle of patients, there is a reduction of active β-catenin, which confirms that the Wnt pathway is altered. Additionally, Yalvac and colleagues [49] already showed in the *CAPN3*-KO model, after generating muscle damage, the altered expression of proteins that are involved in the Akt/mTOR signaling pathway. In muscle tissue from LGMDR1 patients, we also observed alterations in this signaling pathway, since they present a severe decrease in mTOR expression, its phosphorylation, as well as a reduction in the activation of downstream proteins of the pathway, which leads to the dysregulation of the growth of muscle fiber. This signaling pathway, like many others, receives different stimuli through growth factors, cellular energy levels, etc. that must be correctly integrated to stimulate protein synthesis and cell growth according to physiological requirements [50,51,52]. Although little is known about the way in which this integration for an adequate cellular response occurs, [53] demonstrated that TSC2, a critical upstream regulator of mTOR, is responsible for the integration of the signal from the pathway of Wnt and the energy state of the cell.

On one hand, it has been shown that GSK-3β inhibits the mTOR pathway by phosphorylating TSC2, and thus, inhibits the downstream phosphorylation of S6K. Stimulation of the mTOR pathway by Wnt is independent of β-catenin-mediated transcription. The Wnt pathway bifurcates at the level of GSK-3β to regulate, on the one hand, translation by means of mTOR and, on the other hand, transcription by means of β-catenin [53]. Inhibition of GSK-3β has also been confirmed to stimulate the mTOR pathway in the heart during ischemia/reperfusion [54]. In our study, a significant reduction in the activity of the mTOR pathway was observed in muscle tissue from LGMDR1 patients due to the reduced amount of the mTOR protein and its phosphorylation.

Regarding the energy requirement of the cell, AMPK, AMP-activated protein kinase, is a cellular energy sensor that plays an important role in cell homeostasis [55]. Like GSK-3β, AMPK can phosphorylate TSC2, which leads to a negative regulation of mTOR [49,56,57]. Thus, the reduced activity of the mTOR signaling pathway could also be due to the overexpression of AMPK observed in the muscle of the patients. However, the possible changes in AMPK phosphorylation could not be established due to the lack of detection of the phosphorylated protein in muscle samples.

Related to protein expression in pseudo-asymptomatic patients, we observed an increase in most of the analyzed proteins. This fact could be due to the expression profile derived from their young age, since pseudo-asymptomatic patients are 13 and 14 years old and it is known that age is a factor for regulating the expression of genes. Finally, the fact that the muscle samples from pseudo-asymptomatic patients present levels of phosphorylation of p70S6K and RPS6 similar to that of muscles of controls highlights the relevance of the activity of this pathway for the correct maintaining of the muscle function.

### 3.2. Reactivation of the Wnt Pathway and Akt/mTOR Pathway

With the administration of tideglusib and VP0.7 in myotubes, there is a total inhibition of GSK-3β that increases the amount of β-catenin and activates the mTOR pathway, confirming the participation of GSK-3β in both processes. Since the in vitro data shown here have been obtained from a patient and a control, these should be treated with caution. However, this finding supports the suitability of these drugs to be used as a treatment in LGMDR1, given that it could suggest that the undesirable side effects would be low.

Finally, thanks to the results obtained with the *FRZB* gene silencing, it has been established that the GSK-3β pool involved in the Wnt pathway (dependent on Wnt1, Wnt5a, Wnt8 and Wnt9a) does not participate in the regulation of the mTOR pathway. This is because Wnt does not affect the phosphorylation state of GSK-3β [58] and because not all GSK-3β molecules are located in the destruction complex [59].

### 3.3. Lack of Effect of the Treatments on Fibroblasts and CD56-

In the case of Tideglusib and VP0.7, their inhibition is not occurring by the phosphorylation of Ser9 and it cannot be confirmed this way. However, the effect of these drugs at the used concentrations have been confirmed in other experiments [60,61]. Moreover, we consider that, having observed an effect with these doses in myotubes, it was adequate for fibroblasts and CD56-.

Although the results seem promising, they should be treated with caution since they are results that were obtained in vitro and in a single patient. However, this work could help to shed some light on the knowledge of the underlying mechanisms of the LGMDR1 disease. The rescue of signaling pathways responsible of the synthesis of proteins that conform the costamere could help to improve its altered structure. Thus, it could also lead to a better functioning of the muscle fiber, improving the contraction and preventing the degeneration and, consequently, the death of the fiber.

In summary, we established the following relevant events that could orient towards possible therapeutic targets: (a) Wnt and mTOR signaling pathways are altered in the muscles of LGMDR1 patients. (b) Maintaining active the mTOR pathway, pseudo-asymptomatic patients could escape the dystrophic phenotype. (c) *FRZB* gene silencing, through the inhibition exerted on the GSK-3β pool of the Wnt pathway, cannot recover the mTOR pathway. Finally, (d) tideglusib and VP0.7, through efficient inhibition of GSK-3β, rescue the Wnt and mTOR pathways in vitro in the myotubes of LGMDR1 patients.

There are no other approaches in progress for this disease and these treatments could help correct some of the deficits observed in patients. Moreover, tideglusib has shown to be a safe compound for chronic treatments in phase 2 trials [62,63,64,65]. Accordingly, our study supports that the use of tideglusib or VP0.7 could be postulated as possible and safe therapeutic approach to increase protein synthesis that is reduced in LGMDR1 patients. Thus, this treatment would help to avoid the destruction of the structure of the myofibril and to stop the muscular degeneration that patients who suffer from this rare disease undergo.

## 4. Materials and Methods

### 4.1. Muscle Biopsy Samples and Primary Cell Culture

All participants gave informed consent, using forms approved by the Ethics Committee on the Use of Human Subjects in Research at Donostia University Hospital. Muscle biopsy specimens were obtained from adult and pseudo-asymptomatic (05, 09 and 07-109) patients with LGMDR1, in whom the diagnosis had been confirmed genetically by the identification of both mutations in the calpain 3 gene, and from healthy adult controls. These controls were otherwise healthy individuals that underwent surgery for bone fractures and the muscle biopsies were obtained during the surgery (Table 1). The pseudo-asymptomatic patients only showed pathological features in the biopsies. Patient 05: Inflammatory reaction around necrotic and non-necrotic fibers. Inflammation collects at the endomysial site sometimes with perivascular infiltrate without destruction of walls of arterioles and venules. Numerous eosinophilic leucocytes are present. Patient 09: Myositis with local infiltration of eosinophils. Patchy, focal inflammatory cells infiltrate with minor changes in the architecture of fibers without changes in the distribution of the fiber type. Patient 07-109: Myositis with local infiltration of eosinophils. General architecture of fibers is preserved, but focally, fiber destruction and infiltration of eosinophils is observed.

Human proximal muscle biopsies were minced and cultured in a monolayer according to the method described by Askanas [66]. To obtain highly purified myoblasts (CD56+) and CD56- cells, primary cultures were sorted by immunomagnetic selection based on the presence of the early cell surface marker CD56 (separator and reagents from Miltenyi Biotec, Bergisch Gladbach, Germany).

CD56+ cells were seeded at 2500–3000 cells/cm^2^ in culture medium for the myoblast stage. When the myoblasts started to fuse, the medium was replaced by a medium containing 2% of fetal bovine serum (FBS) (Gibco, Thermo Fisher Scientific, Waltham, MA, USA) and without growth factors to obtain myotubes. CD56- cells were seeded in collagen-coated plates.

### 4.2. Fibroblast Isolation and Culture

On collection, skin samples were immersed in RPMI 1640 medium and 2% penicillin (1000 U/mL)/streptomycin (10,000 µg/µL) (Gibco, Thermo Fisher Scientific). Then, skin fragments were placed on a moistened surface with Modified Eagle Medium (MEM) (Gibco, Thermo Fisher Scientific), 13% Newborn Calf Serum (Gibco, Thermo Fisher Scientific), 0.4% penicillin/streptomycin and 2 mM L-Glutamine (Sigma-Aldrich, San Luis, MO, USA) and incubated at 37 °C, with 5% CO_2_. Subsequently, fibroblasts were cultivated in DMEM, 10% FBS and 2% penicillin/streptomycin.

### 4.3. RNA Interference Knockdown

The siRNA for *FRZB* (s5369) knockdown was purchased from Thermo Fisher Scientific. A scrambled siRNA was used as a negative control (AM4611, Thermo Fisher Scientific). Cells plated at 24,000 cells/cm^2^ were transfected with the siRNA at a concentration of 5 nM using RiboCellin transfection reagent (Eurobio, Les Ulis, France) following the manufacturer’s instructions. After 8 days of differentiation, human primary myotubes were incubated with the corresponding siRNA and the transfection agent. Finally, the RNA obtained from these cultures was analyzed 48 h post-transfection by quantitative real-time PCR. Likewise, proteins from these cultures were analyzed 72 h post-transfection.

### 4.4. Administration of GSK-3β Inhibitors

Tideglusib and VP0.7 were synthetized in or laboratories followed previously described procedures [27,29]. Compounds were administered at three different doses (1.2, 3.0 and 15.0 µM). LiCl was purchased from Sigma Aldrich (L7026), San Luis, MO, USA and was administered at a dose of 10 mM. The treatments were added to myotubes at differentiation day 8, as well as to skin fibroblasts and CD56- cells. DMSO (Thermo Fisher Scientific, BP-231) was used as a negative control for Tideglusib and VP0.7. After 48 h of treatment, RNA and proteins were extracted.

### 4.5. RNA Extraction from Myoblast/Myotubes and Muscle Biopsies

RNA extraction from primary myoblast/myotubes was performed with a RNeasy Mini Kit (Qiagen, Hilden, Germany). In the case of the muscle biopsies, which were snap frozen and stored at −80 °C until use, total RNA was obtained using an RNA-Plus Kit (QBiogene, Carlsbad, CA, USA).

### 4.6. Quantitative Real-Time PCR

The isolated RNA was reverse-transcribed to first-strand complementary DNA (cDNA) in a final volume of 50 μL using a High-Capacity cDNA Reverse Transcription Kit (Applied Biosystems, Thermo Fisher Scientific), according to the manufacturer’s instructions. To investigate the levels of expression of the differentially expressed genes (*CAPN3*, *FOS*, *ITGB1BP2*, *ANOS1* and *GAPDH*, Table S1) in these tissues, TaqMan quantitative RT–PCR assays were performed, using the ‘CFX384 Real-Time PCR Detection System’ (Bio-Rad, Hercules, CA, USA). These genes were selected because they are deregulated in the muscle of LGMDR1 patients and their expression was recovered by means of the silencing of *FRZB* gene and the lithium treatment in a previous study [24].

### 4.7. Muscle Tissue and Cell Preparation for Western Blot Analysis

Protein obtention from muscle tissues and cell cultures, as well as the procedure for western blot analysis were performed as previously described elsewhere [24]. The used antibodies are detailed in Table S2. The anti-active-β-catenin antibody is specific for the active form of β-catenin, dephosphorylated on Ser37 or Thr41 [67].

Some limitations have been found in the detection and quantification of different proteins. Total RPS6 bands could not be detected in muscle and myotubes, even though several trials were carried out. P-p70S6K(T389)/p70S6K could not be represented because available samples did not match to obtain the ratio. When a single sample was available, statistical analysis was not performed.

## Figures and Tables

**Table 1:** Muscle and myoblast samples from healthy controls and LGMDR1 patients.

Biopsy Number	Status	Gender	Sample (Tissue of Origin)	Age	Functional Status	*CAPN3* Gene Mutations	
						Mutation 1	Mutation 2
Muscle samples							
27	Control	Male	Quadriceps	50	-	-	-
31	Control	Male	Quadriceps	46	-	-	-
33	Control	Male	Deltoid	51	-	-	-
38	Control	Male	Quadriceps	31	-	-	-
39	Control	Male	Quadriceps	41	-	-	-
42	Control	Female	Quadriceps	42	-	-	-
05	LGMDR1	Male	Deltoid	13	Pseudo- Asymptomatic	p.(Arg788SerfsX14)	p.(Arg788SerfsX14)
09	LGMDR1	Female	Biceps	14	Pseudo- Asymptomatic	p.(Arg490Trp)	p.(Gly691TrpfsX7)
07-109	LGMDR1	Male	*	10	Pseudo- Asymptomatic	p.(Arg698Gly)	p.(Arg748Glu)
36 **	LGMDR1	Male	Quadriceps	26	Ambulant	p.(Lys254Glu)	p.(Pro637HisfsX25)
B10-61	LGMDR1	Female	Quadriceps	23	Ambulant	p.(Pro22Glnfs*35)	p.(Lys211_Glu323del)
B09-26	LGMDR1	Female	Quadriceps	48	Non-ambulant	p.(Arg489Tyr)	p.(Arg788Ser)
97-114	LGMDR1	Male	Deltoid	49	Ambulant	p.(Pro637HisfsX25)	p.(Asp665LeufsX18)
97-168	LGMDR1	Male	*	*	Ambulant	p.(Ser479Gly)	p.(Asp665LeufsX18)
97-169	LGMDR1	Male	Deltoid	51	Ambulant	p.(Ser479Gly)	p.(Asp665LeufsX18)
Myoblast and CD56- cells samples							
09-23	Control	Male	Triceps	26	-	-	-
10-36	Control	Male	Biceps	23	-	-	-
13-05	Control	Male	Quadriceps	14	-	-	-
13-07	Control	Female	Biceps	36	-	-	-
15-12	Control	Male	Deltoid	36	-	-	-
09-21	LGMDR1	Male	Biceps	19	Ambulant	p.(His690ArgfsX9)	p.(His690ArgfsX9)
09-24	LGMDR1	Female	Deltoid	47	Non-ambulant	p.(Arg788SerfsX14)	p.(Lys595ValfsX70)
09-25	LGMDR1	Male	Deltoid	28	Ambulant	p.(Lys254Glu)	p.(Pro637HisfsX25)
10-39	LGMDR1	Male	Deltoid	29	Non-ambulant	p.(Lys254del)	p.(X822Leuext62X)
Skin fibroblasts samples							
F-08-19	Control	Female	Skin	52	-	-	-
F-08-21	Control	Male	Skin	49	-	-	-
F-08-30	Control	Female	Skin	31	-	-	-
F-09-26	LGMDR1	Female	Skin	49	Non-ambulant	p.(Arg489Tyr)	p.(Arg788Ser)
F-09-50	LGMDR1	Female	Skin	67	Non-ambulant	p.(Arg788SerfsX14)	p.(Leu212_Val344delfs *)
F-17-28	LGMDR1	Male	Skin	29	Ambulant	p.(Lys254del)	p.(Arg490Trp)

* information not available. ** 36, also identified as 09-25 in different blots.

## Data Availability

Data are contained within the article and Supplementary Materials.

## Supplementary Materials

The following are available online at https://www.mdpi.com/article/10.3390/ijms22147367/s1.
