# Supplementary material for: Allosteric Modulation of GSK-3β as a New Therapeutic Approach in Limb Girdle Muscular Dystrophy R1 Calpain 3-Related
Source: Int J Mol Sci. 2021 Jul 8;22(14):7367. doi: 10.3390/ijms22147367 (PMC8308041; doi:10.3390/ijms22147367)
Supplement: Supplementary file 1 [file ijms-22-07367-s001.zip › ijms-1255136-supplementary.pdf]

Table S1. Used *TaqMan®* probes

Gene and exon in which the probe binds	Probe code
CAPN3 Ex1-2	Hs00181057_m1
FOS Ex1-2	Hs99999140_m1
ITGB1BP2 Ex4-5	Hs00183746_m1
ANOS1 Ex2-3	Hs01085107_m1
GAPDH Ex2	Hs99999905_m1

Table S2. Used antibody list

Primary Antibody	Manufacturer	Reference
GAPDH	Cell Signaling Technology	#2118
β-catenin	Santa Cruz Biotechnology	SC-7963
GSK-3β	Cell Signaling Technology	#9315
Phospho-GSK-3β (Ser9)	Cell Signaling Technology	#9323
Active-β-Catenin	Milipore	05-665
Akt	Cell Signaling Technology	#9272
Phospho-Akt (Ser473)	Cell Signaling Technology	#4060
mTOR	Cell Signaling Technology	#2983
Phospho-mTOR (Ser2448) [Cells]	Cell Signaling Technology	#2971
Phospho-mTOR (Ser2448) [Muscle]	Cell Signaling Technology	#5536
Phospho-mTOR (Ser2481)	Cell Signaling Technology	#2974
p70 S6 Kinase	Cell Signaling Technology	#2708
Phospho-p70 S6 Kinase (Thr421/Ser424)	Cell Signaling Technology	#9204
Phospho-p70 S6 Kinase (Thr389) [Cells]	Cell Signaling Technology	#9205
Phospho-p70 S6 Kinase (Thr389) [Muscle]	Cell Signaling Technology	#9234
Phospho-S6 Ribosomal Protein (Ser235/236)	Cell Signaling Technology	#2211
AMPKα	Cell Signaling Technology	#2532
Integrin β1D	Milipore	MAB1900
Melusin	Abcam	SC-133780
Phospho-AMPKα (Thr172)	Cell Signaling Technology	#2531

Secondary Antibody	Manufacturer	Reference
Anti-rabbit IgG, HRP-linked	Cell Signaling Technology	#7074
Anti-mouse IgG, HRP-linked	DAKO	P0260

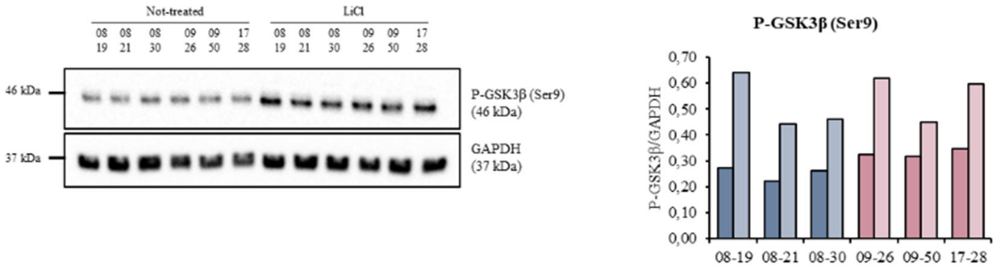

Figure S1. Análisis del efecto en fibroblastos con tratamiento con Li (P-GSK3β en Ser9)

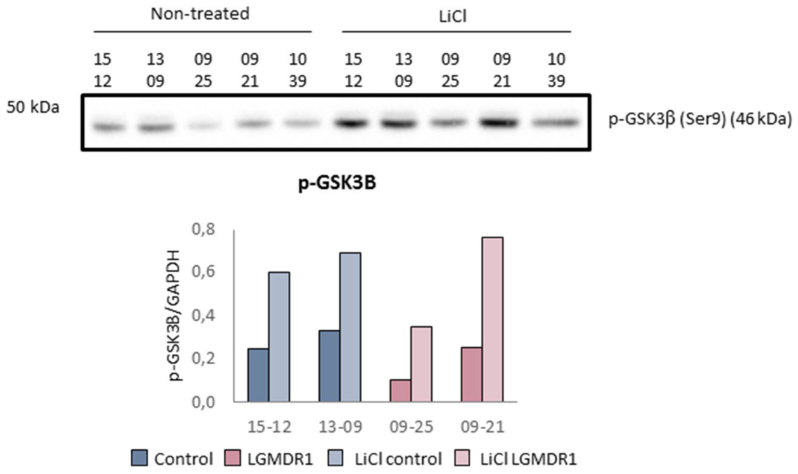

Figure S2. Análisis del efecto en CD56- con tratamiento con Li (P-GSK3β en Ser9)
